# Channelrhodopsin-2 Localised to the Axon Initial Segment

**DOI:** 10.1371/journal.pone.0013761

**Published:** 2010-10-29

**Authors:** Matthew S. Grubb, Juan Burrone

**Affiliations:** Medical Research Council (MRC) Centre for Developmental Neurobiology, King's College London, London, United Kingdom; Claremont Colleges, United States of America

## Abstract

The light-gated cation channel Channelrhodopsin-2 (ChR2) is a powerful and versatile tool for controlling neuronal activity. Currently available versions of ChR2 either distribute uniformly throughout the plasma membrane or are localised specifically to somatodendritic or synaptic domains. Localising ChR2 instead to the axon initial segment (AIS) could prove an extremely useful addition to the optogenetic repertoire, targeting the channel directly to the site of action potential initiation, and limiting depolarisation and associated calcium entry elsewhere in the neuron. Here, we describe a ChR2 construct that we localised specifically to the AIS by adding the ankyrinG-binding loop of voltage-gated sodium channels (Na_v_II-III) to its intracellular terminus. Expression of ChR2-YFP-Na_v_II-III did not significantly affect the passive or active electrical properties of cultured rat hippocampal neurons. However, the tiny ChR2 currents and small membrane depolarisations resulting from AIS targeting meant that optogenetic control of action potential firing with ChR2-YFP-Na_v_II-III was unsuccessful in baseline conditions. We did succeed in stimulating action potentials with light in some ChR2-YFP-Na_v_II-III-expressing neurons, but only when blocking KCNQ voltage-gated potassium channels. We discuss possible alternative approaches to obtaining precise control of neuronal spiking with AIS-targeted optogenetic constructs and propose potential uses for our ChR2-YFP-Na_v_II-III probe where subthreshold modulation of action potential initiation is desirable.

## Introduction

Gaining precise control over neuronal activity in space and time is a major goal in experimental and translational neuroscience. In various model systems, and with increasingly sophisticated technology, attempts to activate endogenous neural tissue have employed electrical [e.g. [1],[2],[3],[4],[5]], magnetic [6] and ultrasound [7], [8] stimulation, with varying degrees of success. Recent years have also seen major advances in means of controlling neuronal activity using light [9]. Photocaged compounds such as neurotransmitters or divalent ions can be used in combination with focal photostimulation to mimic fast, local neuronal signals [10], while synthetically created photoswitch molecules can turn native or genetically altered proteins, including potassium channels [e.g. [11]] and glutamate receptors [e.g.[12]], into light-responsive proteins. Both of these approaches, however, require application of exogenous compounds to neuronal tissue. An alternative strategy is to genetically modify neurons so that they produce inherently light-responsive molecules, an approach known as optogenetics [13] (although optogenetic proteins do require additional chromophores such as all-*trans* retinal to become light-sensitive, these are usually already present in sufficient concentrations in *in vitro* neuronal preparations, and in the mammalian brain). The available range of optogenetic tools is becoming impressively large. We now have means of hyperpolarising neurons by activating chloride pumps with yellow light (halorhodopsin, or (e)NpHR) [14], [15], [16], [17], or by activating proton pumps with yellow or blue-green light (Arch and Mac, respectively) [18]. There are also increasingly sophisticated ways to depolarise neurons, including activation of a cation channel with yellow light (Volvox channelrhodopsin-1, or VChR1) [19], bi-stable excitation that can be switched on with blue light and off with green light (step-function opsins or SFOs) [20], [21], channelrhodopsin variants with improved kinetics (e.g. ChEF) [22], and ultra-fast light-sensitive channels for high-frequency neuronal stimulation (ChETA) [23]. The most intensely studied, best understood and most widely used optogenetic tool, however, is channelrhodopsin-2 (ChR2). ChR2 is a cation channel, isolated from the eyespot of *Chlamydomonas* algae, which is directly opened by illumination with a sufficient intensity of blue light (peak excitation ∼460 nm) [24]. When expressed in vertebrate neurons, it mobilises efficiently to the plasma membrane, and upon light stimulation can produce rapid, large currents carried predominantly by sodium, potassium and calcium ions [24], [25], [26]. Activating these conductances under resting physiological conditions depolarises the neuronal membrane, so if the light stimulus is sufficiently strong, photostimulation combined with ChR2 expression can be used to trigger action potentials with millisecond resolution [e.g. [25],[26]]. Amongst a plethora of current applications, ChR2-induced firing has now been employed *in vitro* and *in vivo* to map neuronal connectivity [27], [28], [29], [30], [31], [32], [33], [34], to induce synaptic plasticity [35], [36], to describe the activity patterns needed for homeostatic plasticity [37], to demonstrate the formation of functional outputs [38], to alter behaviour [39], [40], [41], [42], [43], [44], [45], [46], and even to restore function after spinal cord injury [47] or retinal degeneration [48,49,50, but see 51].

A vital component in determining spiking behaviour in neurons, whether ChR2-driven or not, is a highly-specialised structure known as the axon initial segment, or AIS. As its name suggests, the AIS is located near the start of the axon, where it comprises a complex arrangement of specialised scaffolding proteins, signalling molecules and ion channels [52], [53], [54], [55]. This dense meshwork of interconnected proteins provides a barrier for molecular movement both within the cell membrane [56] and within the cytoplasm [57], making the AIS crucial in regulating axonal transport, and a key player in determining the boundary between axonal and somatodendritic neuronal compartments [55], [58], [59], [60]. In terms of electrical activity in the nervous system, however, the AIS plays a different, vital role. Electrophysiological recordings [e.g. [61],[62],[63],[64],[65],[66],[67],[68],[69],[70]], mathematical modelling approaches [e.g. [68],[69],[71],[72],[73]] and experiments using voltage [70], [74], [75] or sodium [68] indicator dyes all point to the AIS as the subcellular location where action potentials initiate. This ‘hotspot’ for neuronal excitability exists by virtue of the AIS's high concentration of voltage-gated sodium channels (VGSCs) [68], [76], and because of the low voltage activation threshold of certain VGSC subtypes found preferentially in its distal region [73], [77]. It may also be a surprisingly plastic functional compartment, with recent data showing not only that the AIS can disassemble rapidly following cellular injury [60], but also that the structure can relocate along the axon [37], or change its length [78] in response to long-term changes in electrical activity.

Basic fluorophore-bound versions of ChR2 distribute indiscriminately throughout the cell membrane, including the AIS [e.g. [26],[79]]. ChR2 can be targeted to specific neuronal subcompartments, however, by creating fusion protein constructs that bind preferentially to molecules found in particular cellular regions. For example, adding the myosin-Va binding domain (MBD) of melanophilin to the C-terminus of ChR2-YFP produces somatodendritic localisation, with ChR2-YFP-MBD largely excluded from the axon [79]. Fusing the PDZ binding motif ETQV to ChR2-YFP, on the other hand, targets the ion channel preferentially to the postsynaptic side of glutamatergic synapses [80]. So far, though, there are no versions of ChR2 that localise to the AIS, despite the obvious theoretical benefits of an AIS-targeted optogenetic tool. Since action potentials initiate at the AIS, being able to control neuronal depolarisation (or hyperpolarisation) specifically at that initiation site should allow precise temporal control over firing behaviour. Action potentials are triggered at the AIS because of its high concentration of low-threshold voltage-gated sodium channels [68], [73], [76], so targeting ChR2 to this most excitable portion of the cell might also allow for more efficient control over spiking, using lower light stimulus intensities. Finally, for applications where accurate spike triggering is the only goal, localising ChR2 to the AIS avoids any complications associated with depolarisation in the soma and dendrites. This is particularly relevant because ChR2 is permeable to calcium [24], meaning that strong whole-cell photostimulation could lead to significant elevations in somatodendritic [Ca^2^]_i_, and subsequently trigger a range of unwanted intracellular signalling cascades [81].

Localising ChR2 to the AIS might therefore prove extremely useful. But how could it be achieved? An obvious approach is to co-opt strategies already used by native AIS-localised proteins. For example, cell adhesion molecules such as neurofascin (NF)-186 localise to the AIS by binding to the scaffolding protein ankyrin-G, an interaction which depends upon a C-terminus FIGQY domain [82], [83], [84]. Better understood, however, are the AIS-targeting mechanisms of ion channels, particularly VGSCs. Sodium channel subunits Na_v_1.1, 1.2, 1.3 and 1.6 share an ankyrinG binding motif, critically dependent upon a 9 amino acid sequence, which is located in the intracellular loop between their second and third transmembrane domains (II-III loop) [84], [85], [86], [87]. A homologous motif is also found in KCNQ2/3-type potassium channel subunits, which, along with VGSCs, bind to ankyrinG and localise to the AIS [88], [89]. The ankyrinG binding domain alone, however, is not sufficient for AIS targeting of VGSC subunits [87], which also use a removal sequence in their II-III intracellular loop to ensure that they are not stably inserted in the membrane elsewhere in the neuron [87], and several phosphorylation target sites for protein kinase CK2, also in the II-III loop, to regulate ankyrinG binding [90], [91]. Since all of these necessary components for AIS targeting are found in the II-III intracellular loop of all Na_v_1.1, 1.2, 1.3 and 1.6 VGSC subunits [91], it would be no surprise if the Na_v_II-III loop sequence from any of these subunits were sufficient to target novel proteins to the AIS. This is indeed the case: fusing the II-III loop from Na_v_1.2 to a CD4 extracellular domain [86], or the extracellular domain of neurofascin [84], GFP [86] or YFP [37] produces reliable AIS localisation in dissociated hippocampal cultures.

Here we undertook a similar strategy for ChR2, successfully localising ChR2-YFP to the AIS by fusing it to the II-III loop sequence of Na_v_1.2. This construct was expressed in dissociated hippocampal neurons without significantly altering their electrical properties. However, the extremely low magnitude light-evoked depolarisations resulting from its restriction to the AIS meant that we were unable to induce light-evoked spikes in cells expressing ChR2-YFP-Na_v_II-III, except under conditions of artificially increased neuronal excitability.

## Methods

### Molecular Biology and Transfections

The ChR2-YFP construct was pLenti-Synapsin-hChR2(H134R)-EYFP-WPRE, a gift from Karl Deisseroth (www.optogenetics.org). We made ChR2-YFP-Na_v_II-III by cloning the II-III intracellular loop sequence of Na_v_1.2 [86] (SSFSSDNLAATDDDNEMNNLQIAVGRMQKGIDFVKRKIREFIQKAFVRKQKALDEIKPLEDLNNKKDSCISNHTTIEIGKDLNYLKDGNGTTSGIGSSVEKYVVDESDYMSFINNPSLTVTVPIALGESDFENLNTEEFSSESDMEESKEKLNATSSSEGSTVDIGAPAEGEQPEAEPEESLEPEACFTEDCVRKFKCCQISIEEGKGKLWWNLRKTCYKX; MWG) into the BsrgI site found at the C-terminal end of YFP in the ChR2-YFP plasmid. The appropriate base pairs were added to complete the YFP sequence before the Na_v_II-III sequence began. The construct was verified with sequencing, and has been submitted for general distribution with Addgene (www.addgene.org).

### Dissociated Hippocampal Cultures

Humane killing for tissue collection conformed to local King's College London ethical approval under the UK Supplementary Code of Practice, The Humane Killing of Animals under Schedule 1 to the Animals (Scientific Procedures) Act 1986. We prepared dissociated mixed neuronal and glial cultures from E 18 Sprague-Dawley rat hippocampi according to standard protocols, as described previously [37]. Briefly, hippocampi were dissected in HBSS, digested in trypsin (Worthington; 0.5 mg/ml; 15 min 37°C), triturated through Pasteur pipettes of increasingly narrow diameter, and plated at ∼240 cells/mm^2^ on glass coverslips pre-coated with PDL (50 µg/ml) and laminin (40 µg/ml). Cultures were incubated (37°C, 6% CO_2_) in a 1∶1 mixture of neurobasal medium plus B27 supplement (2%) and neurobasal medium plus foetal calf serum (2%), with additional glutamax (500 µM) and penicillin-streptomycin (100 µg/ml). At 7 days *in vitro* (DIV) half of this medium was replaced with neurobasal medium plus B27 supplement. Cells were transfected at 7 DIV using lipofectamine2000, and were studied between 9 DIV and 12 DIV. Unless otherwise specified, all culture reagents were from Invitrogen, UK.

### Immunocytochemistry

We fixed cells with 1% paraformaldehyde (TAAB Laboratories, UK; in 3% sucrose, 60 mM PIPES, 25 mM HEPES, 5 mM EGTA, 1 mM MgCl_2_; pH 7.4; 20 min RT), washed in PBS, then blocked and permeabilised in 3% bovine serum albumin (BSA, Sigma) with 0.4% saponin (Sigma, UK; 1 h RT). We labelled AISs with a chicken polyclonal anti-βIV-spectrin antibody (a gift from M. Komada, Tokyo Institute of Technology, Japan; 1∶500 in PBS with 3% BSA, 1 h RT), followed by PBS washes, and then an Alexa Fluor goat anti-chicken 568 secondary antibody (Invitrogen, UK; 1∶1000; 1 h RT). Coverslips were then given a final PBS wash, and mounted in Mowiol (Calbiochem, UK).

### Imaging and image analysis

Immunostained coverslips were imaged using an Olympus FluoView 1000 laser scanning confocal microscope coupled to FluoView software. We used an Olympus 40x oil immersion objective (0.8 NA). YFP and Alexa Fluor 568 fluorophores were imaged sequentially using 488 nm and 543 nm lasers, respectively, along with appropriate excitation and emission filters. Z-axis image stacks were acquired (1024×1024 pixels at 2x digital zoom, 0.5 µm z-axis steps), exported as raw 16-bit TIFF files, converted into single maximum intensity z-axis projections, and imported into Matlab (Mathworks, UK) for analysis using a custom-written function [37] that is freely available at www.grubblab.org/Matlab-scripts.php and www.mathworks.com/matlabcentral/fileexchange/28181-ais-quantification. We drew a line profile starting at the soma that extended down the axon, through and past the AIS. For our first measure of AIS localisation, we simply took raw fluorescence values along this line profile for both YFP and βIV-spectrin and calculated the Spearman correlation coefficient *r* between the two samples. For our second measure of AIS localisation, we used measures of the start and end positions of the AIS, as described previously [37]. At each pixel (1 pixel  = 0.155 µm) along the axon line profile, βIV-spectrin fluorescence intensity values were averaged over a 3×3 pixel square centred on the pixel of interest. Averaged profiles were then smoothed using a 40-point (∼6 µm) sliding mean, and normalised between 1 (maximum smoothed fluorescence) and 0 (minimum smoothed fluorescence). AIS start and end positions were obtained at the proximal and distal axonal positions, respectively, where the normalised and smoothed profile declined to 0.33. The AIS region defined by these start and end positions was then used to calculate an AIS localisation index (AISi) for the ChR2 constructs based on normalised, smoothed YFP fluorescence intensities within and outside the AIS: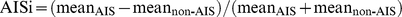 where mean_AIS_ is the mean YFP fluorescence within the AIS region, and mean_non-AIS_ is the mean YFP fluorescence over all points outside the AIS region.

### Electrophysiology

As previously described [37], we made visually targeted whole-cell patch-clamp recordings from cultures maintained in a basic HEPES-buffered saline external solution (pH 7.4, ∼280 mOsm, ∼23°C) that contained, in mM: 136 NaCl, 2.5 KCl, 10 HEPES, 10 d-glucose, 2 CaCl_2_, 1.3 MgCl_2_. Pipettes were pulled from borosilicate glass (OD 1.5 mm, ID 1.17 mm, Harvard Apparatus, Edenbridge, UK), with a resistance of 3–7 MΩ, and were filled with an internal solution containing, in mM: 130 K-gluconate, 10 NaCl, 1 EGTA, 0.133 CaCl_2_, 2 MgCl_2_, 10 HEPES, 3.5 NaATP, 1 NaGTP. Recordings were obtained with a Heka EPC10/2 amplifier coupled to Pulse acquisition software. Signals were Bessel filtered at 10 kHz (filter 1), and 2.9 kHz (filter 2), digitised, and sampled at 25–50 kHz (20–40 µs sample interval). Fast capacitance was compensated in the On-cell configuration.

Electrophysiological parameters were analysed using custom routines written in Matlab. The resting membrane potential (V_rest_) was estimated immediately following membrane patch rupture in current clamp mode with zero holding current (*I* = 0) and, like all voltages in this paper, was adjusted for an estimated liquid junction potential of ∼+15 mV. With slow capacitance compensation inactive in voltage-clamp mode, we used responses to a 10 mV hyperpolarisation step to estimate the neuron's membrane resistance (R_m_; from the steady holding current at the new voltage) and membrane capacitance (C_m_; from the area under the exponentially-decaying current from peak to holding).

Voltage-gated sodium currents were measured in basic HBS external medium (see above) with the addition of 10 mM tetraethylammonium (TEA; Alfa Aesar, UK) to block voltage-gated potassium currents, 100 µM CdCl_2_ (Sigma, UK) to block voltage-gated calcium currents, 10 µM SR-95531 (gabazine; Sigma, UK) to block GABA_A_ receptors, 20 µM 6-Cyano-7-nitroquinoxaline-2,3-dione (CNQX; Sigma, UK) to block AMPA receptors, and 25 µM dl-2-amino-5-phosphonovaleric acid (APV, Sigma, UK) to block NMDA receptors. Potassium conductances were additionally blocked with a Cs-gluconate-based internal solution which contained, in mM: 130 Cs-gluconate, 10 NaCl, 1 EGTA, 0.133 CaCl_2_, 2 MgCl_2_, 10 HEPES, 3.5 NaATP, 1 NaGTP. For these recordings, series resistance was compensated at ≥50% (10 µs). Peak transient sodium currents were measured after subtraction of scaled passive current responses to the appropriate voltage steps. Persistent sodium currents were estimated from the mean whole-cell current over the final 50 ms of prolonged 500 ms membrane potential steps. To compensate for passive current contributions the initial portion of the resulting IV curve was fitted with a regression line. Subtracting this line from the raw data points then gave an estimate of persistent sodium current magnitude.

Spiking behaviour in response to 500 ms somatic current injections was recorded in current-clamp mode with a holding voltage of −75 mV, using the basic HBS external and K-gluconate-based internal solutions described above. Spikes were counted as transient depolarisations passing 0 mV, current threshold (*I*_thresh_) was measured from the lowest current input that reliably evoked at least one spike, and voltage threshold (V_thresh_) was measured as the voltage at which the first derivative of the recording (*dV/dt*) exceeded 10x the standard deviation of its baseline.

Responses of ChR2-expressing neurons to photostimulation in on-cell or current-clamp modes were recorded using the basic HBS external and K-gluconate-based internal solutions described above, with the addition of 10 µM gabazine, 10 µM CNQX and 25 µM APV to block synaptic conductances extracellularly. In some recordings, 100 µM Xe 991 and 100 µM linopirdine (Tocris, UK) were added to the external medium to block KCNQ channels. Whole-cell ChR2 currents were measured in voltage-clamp (−75 mV) using the Cs-gluconate-based internal solution and basic HBS external solution supplemented with 10 mM TEA, 1 µM tetrodotoxin (TTX; Alomone Labs, Israel), and 100 µM CdCl_2_ to block all main voltage-gated channels, and 10 µM gabazine, 10 µM CNQX and 25 µM APV to block all main synaptic conductances. ∼488 nm photostimulation at ∼170 mW/mm^2^ was provided by a shutter-controlled Xenon-arc lamp (Lambda-LS, Sutter Instruments, UK) with appropriate excitation filters (Chroma Tech. Corp., USA) but no neutral density filtering. More powerful ∼1 mW photostimulation was provided by a near-violet diode 405 nm laser controlled by an Olympus FluoView 1000 confocal microscope.

### Statistics

Statistical analysis was carried out in Prism (GraphPad, USA). All tests were two-tailed, with the level of significance (α) set at 0.05.

## Results

**Figure 1:**
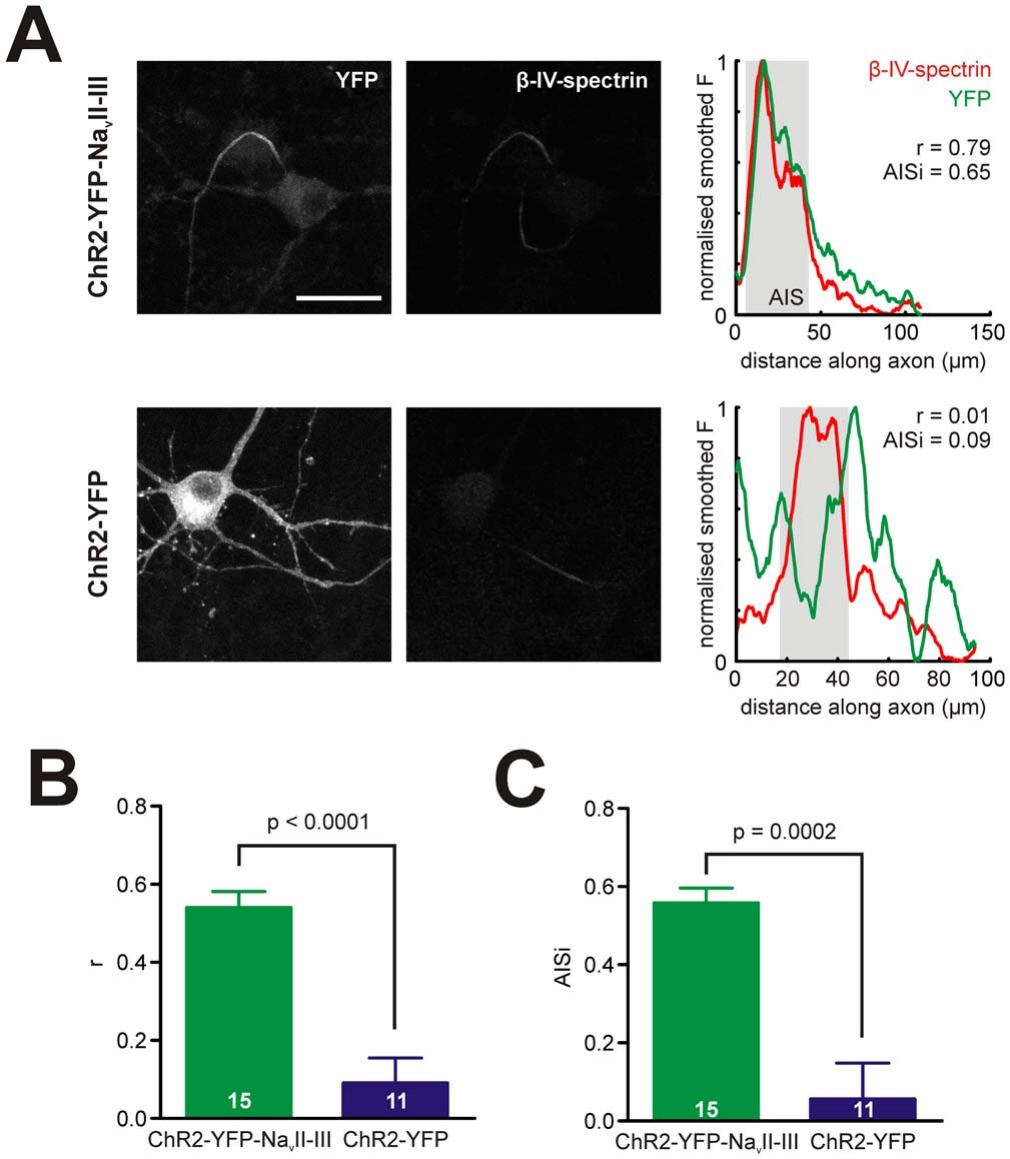
The sodium channel loop Na_v_II-III targets ChR2 to the AIS. A) Example images (left) of neurons expressing ChR2-YFP-Na_v_II-III (top, note background fluorescence revealing the soma of a non-transfected neuron in the YFP channel, with this cell's AIS also clear in the βIV-spectrin channel), or ChR2-YFP (bottom), co-stained for βIV-spectrin. Scalebar, 25 µm. Plots (right) show normalised YFP and βIV-spectrin fluorescence along the axon, with associated AIS localisation indices. Grey box shows AIS location. B, C) Mean correlation *r* (B) between YFP and βIV-spectrin fluorescence, and mean AISi (C; see text) showing AIS localisation of ChR2-YFP-Na_v_II-III. Numbers in bars show *n* for each group; error bars show SEM; p values report results of t-tests.

We targeted ChR2-YFP to the AIS by adding the Na_v_1.2 ankyrinG-binding loop Na_v_II-III [84], [86] to its intracellular terminus. When expressed in cultured hippocampal neurons, the resulting fusion protein ChR2-YFP-Na_v_II-III localised extremely well to the AIS (Figure 1A, top), unlike ChR2-YFP (Figure 1A, bottom). We quantified the degree of AIS localisation of these fusion protein constructs in two ways, both using parallel fluorescent immunocytochemical staining for the AIS scaffolding protein βIV-spectrin. In the first, we obtained normalised fluorescence profiles for βIV-spectrin and YFP along the axon (Figure 1A, right), and simply calculated the Spearman correlation coefficient *r* between these distributions as a measure of YFP AIS localisation. Mean *r* was far higher in neurons expressing ChR2-YFP- Na_v_II-III (mean ± SEM, 0.54±0.04, n = 15) than in neurons expressing ChR2-YFP (0.09±0.06, 11; t-test, p<0.0001; Figure 1B). In the second approach, we used the βIV-spectrin fluorescence distribution to define the extent of the AIS (see Methods) [37], and obtained an AIS index (AISi) from the ratio of YFP fluorescence within the AIS region to YFP fluorescence outside the AIS region (see Methods). Mean AISi values were also much greater in ChR2-YFP- Na_v_II-III -expressing neurons (mean ± SEM, 0.56±0.04; n = 15 cells) versus cells expressing ChR2-YFP (0.06±0.09, 11; t-test with Welch's correction for unequal variances, p = 0.0002; Figure 1C). Addition of the ankyrinG-binding loop Na_v_II-III therefore successfully targets ChR2-YFP to the AIS.

**Figure 2:**
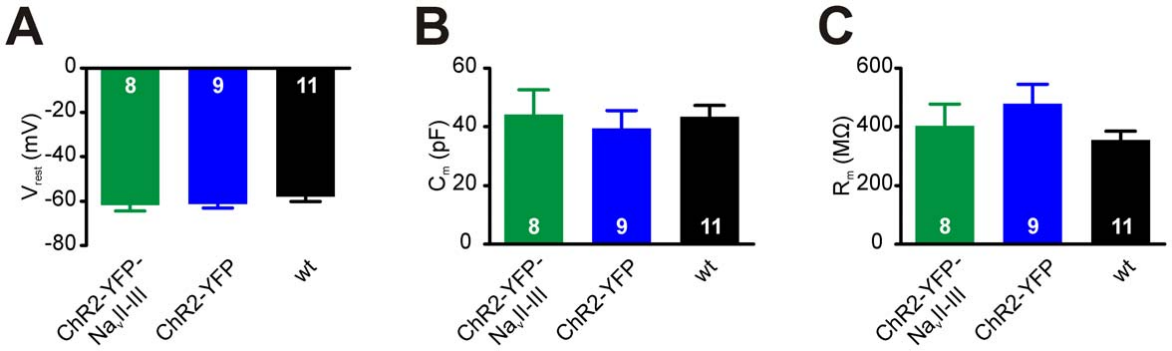
Passive electrical properties of neurons expressing ChR2-YFP-Na_v_II-III. A–C) Mean resting membrane potential (A), cell capacitance (B) and membrane resistance (C) do not differ significantly between ChR2-YFP-Na_v_II-III-positive, ChR2-YFP-positive, or wild-type neurons (1-way ANOVA, p>0.29). Numbers in bars show *n* for each group; error bars show SEM.

We used whole-cell patch-clamp recordings to verify that expressing AIS-targeted ChR2-YFP-Na_v_II-III in neurons did not affect their fundamental electrophysiological properties. Basic functional parameters including the resting membrane potential (V_m_), whole-cell capacitance (C_m_), and passive membrane resistance (R_m_) were not significantly different between neurons expressing ChR2-YFP-Na_v_II-III (V_m_: mean ± SEM, −61.8±2.6 mV; C_m_: 44.2±8.5 pF; R_m_: 403±74 MΩ; n = 8), those expressing ChR2-YFP (V_m_: −61.2±1.8 mV; C_m_: 39.4±6.1 pF; R_m_: 478±67 MΩ; n = 9), and non-transfected wild-type (wt) cells (V_m_: −57.9±2.2 mV; C_m_: 43.4±3.9 pF; R_m_: 354±30 MΩ; n = 11; Figure 2A–C; one-way ANOVA, F_2,27_<1.3, p>0.29).

**Figure 3:**
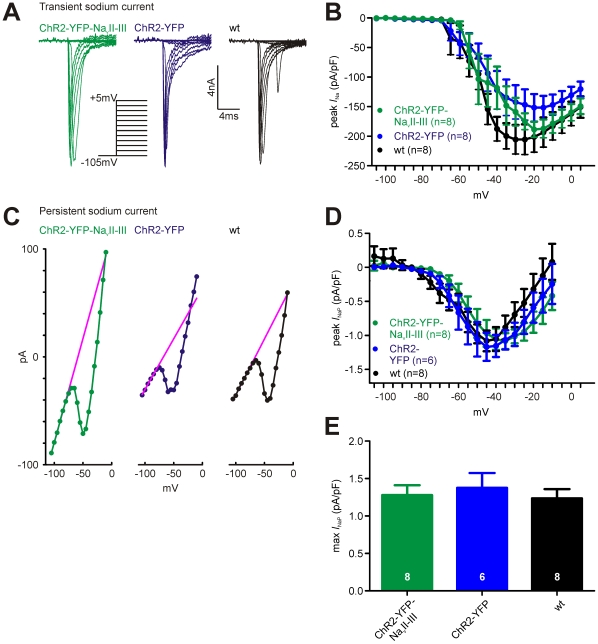
Whole-cell sodium currents in cells expressing ChR2-YFP-Na_v_II-III. A) Example whole-cell transient sodium currents in response to depolarisation steps from −105 mV in ChR2-YFP-Na_v_II-III-expressing, ChR2-YFP-expressing, and wild-type neurons. B) No significant difference in mean whole-cell transient sodium current *I*_Na_ density in the three cell types (effect of cell type in 2-way repeated-measures ANOVA, p = 0.27). Error bars show SEM. C) Example *IV* relationships for whole-cell persistent currents following 500 ms membrane potential steps in the three cell types. The linear fit used to subtract passive currents is shown in magenta. D) No significant difference in mean whole-cell persistent sodium current *I*_NaP_ density across the three cell types (effect of cell type in 2-way repeated-measures ANOVA, p = 0.60). Error bars show SEM. E) No significant difference in maximum persistent sodium current *I*_NaP_ density in the three cell types (one-way ANOVA, p = 0.58). Error bars show SEM.

Because ChR2-YFP-Na_v_II-III uses the same mechanism as native voltage-gated sodium channels to localise to the AIS, we were concerned that ChR2-YFP-Na_v_II-III expression might result in competition for ankyrinG binding sites and a consequent reduction in sodium current magnitude [86]. However, although there was a trend for ChR2-YFP-Na_v_II-III-expressing neurons to have lower voltage-gated transient sodium current densities than wt cells, they displayed a trend towards *larger* transient sodium current densities than ChR2-YFP-expressing neurons, and neither of these trends was statistically significant (Figure 3A,B; repeated-measures 2-way ANOVA: effect of construct, F_2,462_ = 1.39, p = 0.27; effect of voltage, F_22,462_ = 66.75, p<0.0001; effect of interaction, F_44,462_ = 0.94, p = 0.58. Note that a small but significant difference in series resistance (R_s_) was also observed in these recordings; ChR2-YFP-Na_v_II-III mean ± SEM, 10.7±0.8 MΩ, n = 8; wt, 14.2±1.2 MΩ, n = 8; Dunn post-test following Kruskal-Wallis non-parametric one-way ANOVA, p<0.05). Because whole-cell transient sodium current measurements are particularly sensitive to space-clamp errors, we also assessed persistent sodium current (*I*_NaP_) magnitude in cells expressing ChR2-YFP-Na_v_II-III. This current can be clamped well in dendritic cells, and is known to emanate from the AIS [76], [92]. We estimated *I*_NaP_ in conditions of synaptic, calcium and potassium channel blockade by measuring whole-cell currents observed at the end of prolonged 500 ms voltage steps, after subtraction of passive current contributions (see Methods; Figure 3C). The resulting *I*_NaP_ current was entirely blocked by 5 µM TTX (n = 3 wild-type neurons, data not shown). We found no significant differences in *I*_NaP_ magnitude between ChR2-YFP-Na_v_II-III-positive, ChR2-YFP-positive, and wild-type neurons (Figure 3D,E; repeated-measures 2-way ANOVA on *I*_NaP_ current density: effect of construct, F_2,361_ = 0.52, p = 0.60; effect of voltage, F_19,361_ = 26.95, p<0.0001; effect of interaction, F_38,361_ = 1.05, p = 0.40. One-way ANOVA on maximum *I*_NaP_ current density, F_2,21_ = 0.22, p = 0.81).

**Figure 4:**
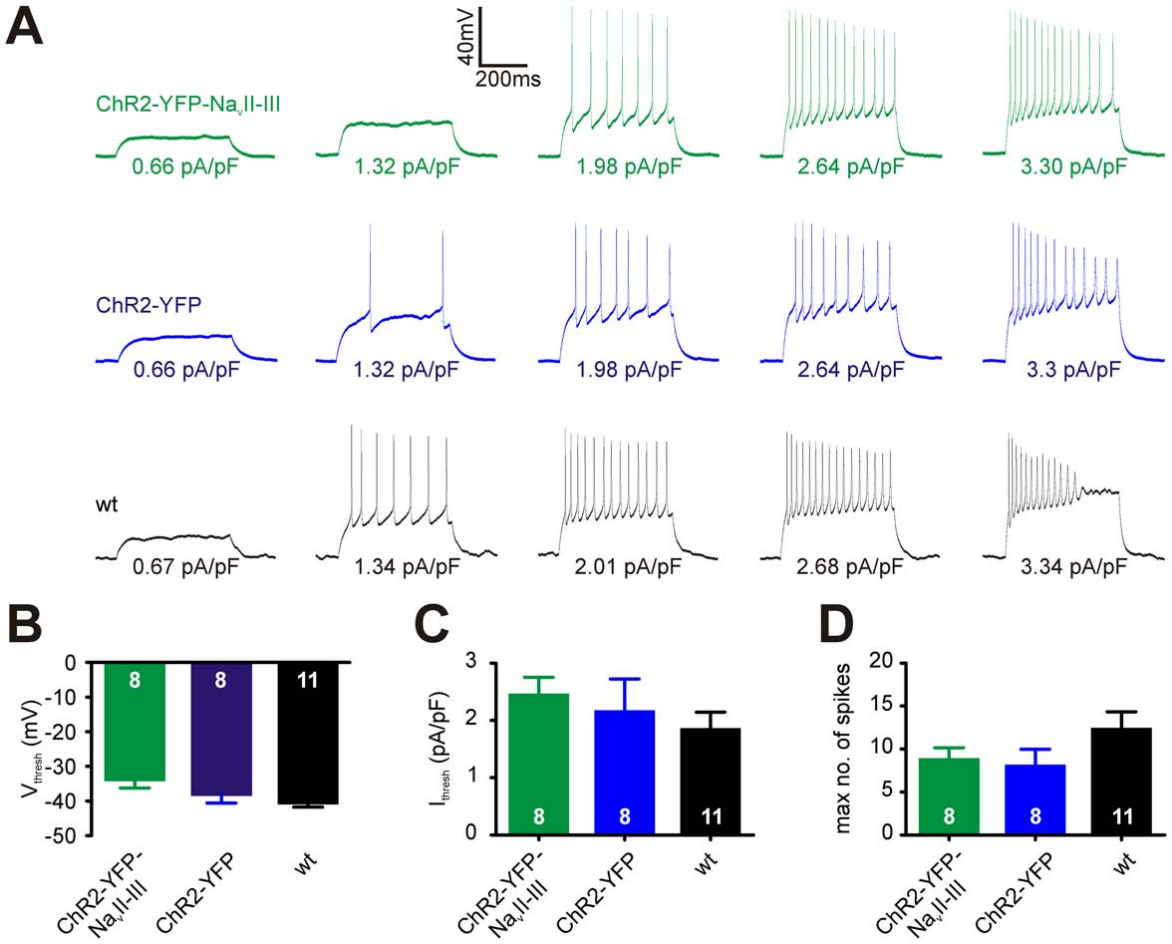
Spiking properties of cells expressing ChR2-YFP-Na_v_II-III. A) Example current-clamp traces in response to 500 ms positive somatic current injection in the ChR2-YFP-Na_v_II-III-expressing, ChR2-YFP-expressing, and wild-type neurons. Values beneath each trace show current injection magnitude in pA/pF. B–D) Mean voltage threshold (B; 1-way ANOVA, p = 0.07), current threshold (C; p = 0.38), and maximum spike number (D; p = 0.24) do not differ significantly across the three cell types. Numbers in bars show *n* for each group; error bars show SEM.

These results were mirrored in measures of action potential firing. Cells transfected with ChR2-YFP-Na_v_II-III could fire spikes in current-clamp mode perfectly well (Figure 4A), and although they showed trends towards depolarised voltage thresholds (Figure 4B; ChR2-YFP-Na_v_II-III mean ± SEM, −33.8±2.4 mV, n = 8; ChR2-YFP, −38.0±2.5 mV, n = 8; wt, −40.5±1.3 mV, n = 11; one-way ANOVA, F_2,26_ = 2.96, p = 0.07), higher current thresholds (Figure 4C; ChR2-YFP-Na_v_II-III mean ± SEM, 2.44±0.31 pA/pF, n = 8; ChR2-YFP, 2.15±0.58 pA/pF, n = 8; wt, 1.84±0.31 pA/pF, n = 11; Kruskal-Wallis non-parametric one-way ANOVA, p = 0.38), and lower maximum spike number (across all current injection magnitudes for a given cell; Figure 4D; ChR2-YFP-Na_v_II-III mean ± SEM, 8.75±1.41, n = 8; ChR2-YFP, 8.00±1.98, n = 8; wt, 12.27±2.08, n = 11; one-way ANOVA, F_2,26_ = 1.51, p = 0.24), none of these trends was statistically significant.

**Figure 5:**
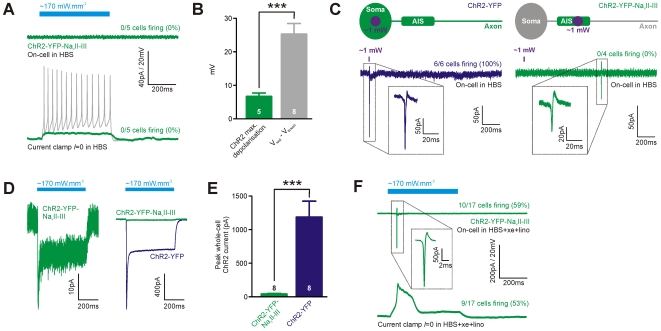
Attempting to control neuronal activity with ChR2-YFP-Na_v_II-III. A) Example on-cell (top) and current-clamp *I* = 0 (bottom) recordings in control HBS solution, showing lack of spiking in a ChR2-YFP-Na_v_II-III-expressing neuron in response to 500 ms blue light stimulation. Grey trace shows spiking in the same cell in response to somatic current injection. B) Mean depolarisation produced by light stimulation in ChR2-YFP-Na_v_II-III-positive cells (current-clamp *I* = 0) is much smaller than the mean depolarisation required to fire an action potential from rest. Numbers in bars show *n* for each group; asterisks show t-test result; ***, p<0.001. Error bars show SEM. C) ChR2-YFP-Na_v_II-III-positive cells fail to fire even to high intensity laser photostimulation. Left, brief ∼1 mW 405 nm laser illumination targeted to the soma of ChR2-positive cells always produced an action potential. Right, the same stimulus at the AIS of ChR2-YFP-Na_v_II-III-positive cells never produced an action potential, even though these cells were capable of firing spontaneous spikes. D) Example whole-cell ChR2 currents in a ChR2-YFP-Na_v_II-III-positive neuron (left) and scaled in comparison to a typical ChR2-YFP-expressing cell (right), recorded at −75 mV in response to 500 ms blue light stimulation. E) Mean peak whole-cell ChR2 current is significantly smaller in ChR2-YFP-Na_v_II-III-positive neurons. Numbers in bars show *n* for each group; asterisks show t-test result; ***, p<0.001. F) Blocking K^+^ channels at the AIS can allow optogenetic control of neuronal activity with ChR2-YFP-Na_v_II-III. Example on-cell (top) and current-clamp *I* = 0 (bottom) traces in HBS supplemented with 100 µM Xe-991 (xe) and 100 µM linopirdine (lino), showing broad spikes in a ChR2-YFP-Na_v_II-III-expressing neuron in response to 500 ms blue light stimulation.

ChR2-YFP-Na_v_II-III therefore localises to the AIS without affecting the basic health or action potential-generating properties of its host cells. But can the construct be used to provide optogenetic control over spiking behaviour? We initially attempted to evoke action potentials in ChR2-YFP-Na_v_II-III-expressing cells using blue light from a Xenon arc fluorescent lamp at maximum intensity (∼170 mW/mm^2^). This light source is routinely used to evoke spikes in ChR2-YFP-positive cells in our laboratory, but even long, 500 ms flashes never produced an action potential in ChR2-YFP-Na_v_II-III-expressing cells (Figure 5A; on-cell and current-clamp *I* = 0 recording configurations, n = 5). As shown above, this was not because ChR2-YFP-Na_v_II-III-positive neurons were unable to fire action potentials *per se* (Figure 5A, grey trace). Instead, the problem was one of ChR2 response magnitude, with the maximum depolarisation produced by light stimulation measured at the soma (mean ± SEM, 6.72±0.99 mV) being ∼20 mV smaller than the depolarisation needed to reach spike threshold from rest (25.3±3.15 mV; Figure 5B). We tried to augment light-evoked ChR2-YFP-Na_v_II-III responses by increasing the intensity of our light stimulus, employing focal AIS stimulation with a near-violet diode 405 nm laser (∼1 mW at the coverslip surface). However, although this strong stimulus reliably evoked spikes in ChR2-YFP-positive cells, even when directed to the soma (Figure 5C, left; 6/6 cells), it never evoked an action potential in neurons expressing ChR2-YFP-Na_v_II-III (Figure 5C, right; 0/4 cells). The magnitude of light-evoked responses in ChR2-YFP-Na_v_II-III-positive cells therefore appears limited by the low total number of ChR2 molecules present in the cell membrane. This was evidenced by voltage-clamp recordings of whole-cell ChR2 currents (Figure 5D), which showed that peak light-evoked responses were ∼28 –fold smaller in neurons expressing ChR2-YFP-Na_v_II-III versus those expressing ChR2-YFP (Figure 5E; Xenon lamp stimulation at ∼170 mW/mm^2^; ChR2-YFP-Na_v_II-III mean ± SEM, 42.1±8.4 pA, n = 8; ChR2-YFP, 1188.5±235.3 pA, n = 8). So, whilst it localises extremely well to the AIS, the ChR2-YFP-Na_v_II-III construct simply provides too little light-evoked current to allow optogenetic control of spiking under baseline conditions.

We did, however, succeed in producing light-evoked action potentials in some ChR2-YFP-Na_v_II-III-expressing cells under rather specialised conditions. As well as clustering the voltage-gated sodium channels that are vital for spike initiation, the AIS also contains high densities of voltage-gated potassium channels that limit and control action potential production [e.g. [53]]. Blocking KCNQ potassium channels, which cluster preferentially at the AIS [88], with the antagonists Xe-991 (100 µM) and linopirdine (100 µM) produced broad spikes that were initiated at much less depolarised membrane potentials, making cells significantly more excitable. In a subset of neurons expressing ChR2-YFP-Na_v_II-III (on-cell, 10/17 cells = 59%; current-clamp *I* = 0, 9/17 cells = 53%), this allowed the production of single action potentials using long-duration high-intensity blue light stimulation (Figure 5F). The temporal precision of spike initiation under these conditions was poor, however: mean time-to-spike following light stimulus onset in on-cell mode was 70 ms, with a standard deviation of 37 ms across all ChR2-YFP-Na_v_II-III-expressing cells.

## Discussion

We have shown that the sodium channel Na_v_II-III loop sequence is sufficient to accurately target ChR2 to the AIS in cultured hippocampal neurons. Expressing this construct does not significantly alter the basic functional properties of its host neurons. However, controlling action potential initiation with ChR2-YFP-Na_v_II-III proved impossible, even with strong AIS-directed laser photostimulation. ChR2 currents localised to the AIS were simply too small to take a neuron past spike threshold, except under artificial conditions of increased excitability.

As predicted by previous success with simple fluorophore [37], [86] and membrane-bound [84], [86] molecules, adding the entire sodium channel II-III loop to the intracellular terminus of ChR2-YFP was sufficient to target the protein specifically to the AIS. Attempts at localising ChR2 to the AIS using only the 9 aa [84] or 27 aa [86] ankyrinG binding domains were less successful (data not shown), suggesting that ChR2, like VGSCs, requires the II-III loop membrane removal sequence [87] and/or CK2 phosphorylation sites [90] for complete AIS targeting. These multiple mechanisms of subcellular localisation, however, make it unlikely that the number of ChR2-YFP-Na_v_II-III molecules could be increased at the AIS by simply increasing expression of the protein with, say, stronger promoters or lentiviral infection [e.g. [16],[93]]. Although producing more foreign protein is an obvious approach to the problem of low ChR2 currents and lack of light-induced spiking, the need for ChR2-YFP-Na_v_II-III to be constitutively removed from non-AIS locations [87] means that cells expressing very high levels of the construct would probably display impaired AIS targeting – they would be simply swamped with excess ChR2. Even more pertinent is the fact that ChR2-YFP-Na_v_II-III uses the same mechanisms as native sodium [84], [86], [87], [90] and potassium [88] channels to localise to the AIS. We saw no significant decrease in sodium current density in our ChR2-YFP-Na_v_II-III-expressing neurons, but we did see a trend towards lower transient *I*_Na_ amplitude (Figure 3), while others have reported large decreases in transient sodium currents with strong expression of GFP-Na_v_II-III [86]. Even if hugely-increased densities of ChR2-YFP-Na_v_II-III could be precisely localised to the AIS, then, they might out-compete native VGSCs to such an extent that action potential initiation is impaired and light-evoked spiking remains impossible.

Are there other means by which ChR2 can be localised to the AIS, without competing with native VGSCs? Cell adhesion molecules such as neurofascin-186 bind ankyrinG and become AIS-targeted via an intracellular FIGQY motif [82], [83], [84], so in theory adding this sequence to ChR2-YFP could localise it to the AIS without interfering with sodium channel binding sites. However, although the FIGQY motif is necessary for cell adhesion molecule binding to ankyrinG, it is not known whether it is sufficient for AIS targeting. Similarly, another AIS scaffolding molecule βIV-spectrin requires spectrin repeat 15 for ankyrinG binding and AIS localisation, but a combination of spectrin repeats 14+15 proved insufficient for full AIS targeting of a myc construct [94]. Finally, the clustering of K_v_1 channels at the AIS is dependent upon the scaffolding molecule PSD-93 [95], but the molecular mechanisms governing this interaction are presently unclear. There are many alternative potential means of targeting ChR2 to the AIS, therefore, but none currently understood sufficiently to be able to be put into practice.

Instead, perhaps the answer to controlling spikes with ChR2 at the AIS lies away from the AIS targeting mechanism, and more towards ChR2 itself. Simple cell compartment trafficking sequences added to the ChR2-YFP-Na_v_II-III construct, for example, might increase the density of ChR2 molecules at the AIS and improve optogenetic efficiency [e.g. [17]], but with all of the caveats associated with overexpression outlined above. Alternatively, variants of ChR2 could be utilised that are more sensitive to light, or have improved single-channel conductance, producing larger light-evoked currents with unchanged AIS channel densities. Such variants are a major future target of optogenetic molecular engineering. In the meantime there are slow-kinetic ChR2 mutants which, over time, produce hugely increased integral currents for a given light stimulus [20], [21], and which could prove useful in evoking spikes via AIS-targeted ChR2 expression, so long as low frequency light-evoked firing is the goal.

Finally, rather than attempting full control over spike initiation with AIS-localised ChR2, maybe the most attainable target is to *influence* action potential firing with optogenetic tools localised to the AIS. Rather than initiating spikes with AIS-based optogenetics, action potentials could be specifically inhibited or shaped by light-driven chloride [14], [15], [16], [17] or proton [18] pumps fused to the Na_v_II-III sequence. In its native state, the AIS contains multiple mechanisms for altering the initiation and subsequent properties of action potentials [53], including voltage-gated potassium channels [e.g. [96],[97]], voltage-gated calcium channels [98], [99], sodium channel modulators [100], [101] and synaptic inputs [102], [103], [104], [105]. Returning to the construct described here, perhaps the small photocurrents produced by ChR2-YFP-Na_v_II-III could be used in various experimental settings to mimic, trigger, or influence these mechanisms of action potential modulation.
